# Editorial: Coronavirus Disease (COVID-19): Psychological and Behavioral Consequences of Confinement on Physical Activity, Sedentarism, and Rehabilitation

**DOI:** 10.3389/fpsyg.2022.816368

**Published:** 2022-03-22

**Authors:** Luis Mochizuki, Michael Brach, Pedro L. Almeida, Ricardo De La Vega, Mauricio Garzon, Julia Maria D'Andrea Greve, Margarita Limon

**Affiliations:** Department of Orthopedic and Traumatology, University of São Paulo, São Paulo, Brazil

**Keywords:** Coronavirus, pandemic (COVID-19), lockdown, psychological distress, physical activity

## Introduction

COVID-19 is an unprecedented global crisis and has changed the world. Lockdown, confinement, and other sanitary actions were developed to mitigate this coronavirus spread. Under such unusual conditions, barriers to keep an active lifestyle were hard to overcome. Fifty-nine articles written by authors from Africa, America, Asia, Europe, and Oceania provide a rich discussion about the topic “Coronavirus Disease (COVID-19): Psychological and Behavioral Consequences of Confinement on Physical Activity, Sedentarism, and Rehabilitation.” This collection is dedicated to understanding the adaptive psychological and behavioral responses to COVID-19, with the focus on the consequences of exercise and physical activity programs for different populations, across the lifespan. The COVID-19 context is explored to discuss lockdown and confinement, physical and social distances, fear of diseases, and opportunities to use innovative technologies in health.

## Contributions

Physically active persons [athletes (Abenza-Cano et al.; Anyan et al.; Bazett-Jones et al.; Freire et al.; Fuentes-García et al.; González-Hernández et al.; Li et al.; Lautenbach et al.; Mehrsafar et al.; Rubio et al.; Szczypińska et al.) and active adults (Guicciardi and Pazzona)] were surveyed to ascertain how the pandemic has affected their behavioral, psychological, and training patterns. Training activities were changed, the coach-athlete relation was evaluated, and coping-pandemic issues to deal with stress were discussed. These studies present how athletes in different sports have adapted their activities during confinement due to COVID-19.

Parents had to deal with emotional and behavioral problems of their children induced by the confinement. Monteiro et al. evaluated how parents and children younger than 7 years old behaved under the stress of lockdown, while Ren et al. discussed parenting stress to deal with children with special needs during the pandemic.

Most of the original research studies analyzed the relations of physical activity and different dimensions of human behavior in adults. Therefore, physical activity behavior was compared with eating behavior (Constant et al.; Diniz et al.; Ingram et al.; Machado-Lima et al.), health risk habits (Diniz et al.), mental health (Diniz et al.), motivation to exercise (Leyton-Román et al.), psychological states (Ingram et al.; Hargreaves et al.; León-Zarceño et al.; Machado-Lima et al.; Meira et al.; Sang et al.; Terry et al.), sleep (Ingram et al.), self-efficiency to exercise (Teran-Escobar et al.), and wellbeing (Brand et al.; Jenkins et al.).

Different strategies to manage the issues during the pandemic were also presented. To promote active behaviors, Marchant et al. asked adults about the use of eHealth for exercise and physical activity, Carfora and Catellani assessed persuasive messages to promote physical activity at home, while Alsalhe et al. evaluated the association between physical activity and fear of COVID-19. Abu-Akel et al. compared the effect of who gives information about social distancing on individual behavior. Borrega-Mouquinho et al. compared the effects of high and moderate-intensity training on psychological factors in a randomized controlled trial. Reigal et al. applied a data mining approach to find behavioral patterns. Alsukah et al. investigated the awareness of COVID-19.

Across the lifespan, other original research articles studied the connections between physical activity and behavior. Physical activity levels were also affected in teenagers and young adults. Bösselmann et al. evaluated the relation among physical activity, boredom, and fear of COVID-19. Liébana-Presa et al. studied stress, emotional intelligence, and the intention to use cannabis during the confinement. Young adults were emotionally affected by the pandemic. Wang et al. showed anxiety in Chinese college students. Lippke et al. investigated physical activity, loneliness, and friendship in European university students. Slimani et al. studied the relation between physical activity and quality of life in young adults in Tunisia. In older adults, Carvalho et al. evaluated the effects of confinement in physical fitness and physical activity behavior, and Lage et al. analyzed the association between depressive symptoms and physical activity intensity during the lockdown. While Torriani-Passn et al. evaluated the barriers and facilitators for stroke survivors to engage in a remote physical exercise program, da Silva, da Silva, et al. studied the use of game platforms for home-based telerehabilitation in patients with cerebral palsy.

For the perspective and opinion articles, different proposals were discussed to face the health issues and sedentary behavior during the pandemic. Rüth and Kaspar have highlighted the benefits of exergaming at home, regarding its physical, and non-physical effects. Sá Filho et al. emphasized the importance of the recommendations of exercising and training at home during the pandemic. Aguirre-Loaiza et al. discussed the embodied cognition approach to explain the importance of physical activity during the pandemic. Filipas et al. discussed the differences for athletes from low- and high-income countries to be continuously engaged with their training routines during the pandemic.

The narrative reviews have gathered the impacts of physical activity in health during the pandemic. Ghram et al. discussed the importance of exercising to reduce the deleterious effects of the COVID-19 pandemic. Clemente-Suárez et al. reviewed the impact of physical inactivity and modifications in nutritional habits, at psychological and physiological levels during the pandemic. Scartoni et al. discussed the protective effect of physical exercise against COVID-19 infection in the elderly.

Methods studies have provided interesting new tools to understand the consequences of the COVID-19 pandemic on health and physical activity. da Silva, de Oliveira, et al. proposed a protocol to compare conventional intervention and non-immersive virtual reality rehabilitation in an COVID-19 inpatient hospital unit. Lete-Lasa et al. propose a tool to identify potential COVID-19 contagion situations in sports and physical education situations. Berasategi et al. proposed and validated a scale to measure wellbeing of children in lockdown.

## Summary

This collection shows that physical activity behavior and COVID-19 are related. Active life during the pandemic provided fewer negative psychological states in different populations. Lockdown and social distancing have created conditions to increase stress, which exercising was a positive action for its reduction or prevention.

## Author Contributions

All authors listed have made a substantial, direct, and intellectual contribution to the work and approved it for publication.

## Conflict of Interest

The authors declare that the research was conducted in the absence of any commercial or financial relationships that could be construed as a potential conflict of interest.

## Publisher's Note

All claims expressed in this article are solely those of the authors and do not necessarily represent those of their affiliated organizations, or those of the publisher, the editors and the reviewers. Any product that may be evaluated in this article, or claim that may be made by its manufacturer, is not guaranteed or endorsed by the publisher.

## Supplementary Material

The Supplementary Material for this article can be found online at: https://www.frontiersin.org/articles/10.3389/fpsyg.2022.816368/full#supplementary-material
